# Lymph Node Dissection for Differentiated Thyroid Cancer

**DOI:** 10.4274/2017.26.suppl.02

**Published:** 2017-01-09

**Authors:** Aviram Mizrachi, Ashok R. Shaha

**Affiliations:** 1 Memorial Sloan-Kettering Cancer Center, Head and Neck Service, New York, USA

**Keywords:** thyroid cancer, lymph node metastases, neck dissection

## Abstract

Lymph node metastases in differentiated thyroid cancer (DTC) have a wide spectrum of clinical significance. Several variables are taken under consideration when trying to decide on the optimal management of patients with DTC. Routine prophylactic central and/or lateral lymph node dissection is not advocated with exception of central neck dissection for locally advanced tumors. When regarding recurrent disease, foundations have been laid for clinicians to make accurate decisions as to when to perform surgery and when to continue maintaining the patient’s disease under observation. These complex decisions are determined based upon multiple factors, not only regarding the patient’s disease but also the patient’s comprehension of the procedure and apprehension levels. Nevertheless if the patient and/or clinician are emotionally keen to surgically remove the disease then the procedure should be considered.

## Abstract (tr)

Diferansiye tiroid kanserinde (DTK) lenf nodu metastazının geniş bir yelpazede klinik önemi vardır. DTK’lı hastaların optimal tedavi yönetimine karar verilirken birçok değişken hesaba alınır. Rutin profilaktik santral ve/veya lateral lenf nodu diseksiyonu lokal ileri tümörlerde santral boyun diseksiyonu istisnası dışında savunulmamaktadır. Rekürrens durumunda klinisyenlerin ne zaman ameliyat gerçekleştirmesi ve ne zaman takip edilmesi kararını verebilmeleri için kılavuzlar oluşturulmuştur. Bu karmaşık kararlar genellikle tek bir faktöre dayanarak değil, aksine hastanın anlama ve katılma düzeyleri ile uyum içinde birçok faktör ile dikkate alınarak verilir. Bununla birlikte, hasta ve/veya klinisyen hastalığı cerrahi olarak çıkartmaya ısrarcı ise o zaman bu seçenek düşünülmelidir.

## OVERVIEW

The incidence of thyroid cancer, specifically well differentiated, is rapidly increasing. The Surveillance, Epidemiology and End Results Program has estimated that there will be 62,450 new cases of thyroid cancer and an estimated 1,950 people will die of this disease in 2015. Nevertheless, the overall five-year survival rate for thyroid cancer is almost 98% and has been constant over the last three decades (1).

The majority of differentiated thyroid cancers (DTC) are usually diagnosed at an early stage, during a routine check up or more increasingly as an incidental finding of neck ultrasonography (US). In these cases, the presence of clinically apparent nodal metastases is uncommon. That being said, multiple studies have shown that the incidence of occult lymph node metastases may reach up to 60%, but this microscopic disease has no prognostic value in patients with DTC (2). Several risk factors for the presence of central lymph node metastases in DTC have been previously described and found to be primary tumor size, extra-thyroidal extension (ETE) and aggressive histological subtypes (3). The factors that are most predictive of central lymph node metastases are male sex, young and old age and primary tumor size (4). Special attention should be given to involvement of the pre-laryngeal (Delphian) lymph node in DTC, which is often associated with ETE and increased incidence of central and lateral neck lymph node metastases. Extrapolating from that, surgeons should consider sampling the Delphian lymph node and perform a frozen section as a form of “sentinel lymph node biopsy” that if found positive may warrant further evaluation of the central and lateral nodal compartments (5). The current AJCC staging system (7^th^ edition) makes a distinction between central nodal involvement (N1a) and lateral nodal involvement (N1b). However, in some cases it may be difficult to distinguish between levels VI and VII, which are adjacent and represent different nodal stages with the latter being N1b.

The current approach towards the role of prophylactic neck dissection precludes any survival benefit in patients with a clinically negative neck and may result in unnecessary upstaging that could subject them to radioactive iodine (RAI) treatment (6). Prophylactic central compartment neck dissection (ipsilateral or bilateral) should be considered in patients with DTC with clinically uninvolved central neck lymph nodes (cN0) who have locally advanced primary tumors (T_3_ or T_4_), clinically involved lateral neck nodes (cN1b), or if the information will be used to plan further steps in therapy (7). This approach has reached consensus among surgeons in relation to the lateral neck, while for the central compartment some still advocate a prophylactic lymph node dissection. One argument for this approach is that the central compartment is readily accessible while performing the thyroidectomy and that clearing this compartment during the first surgical procedure is easier and safer than in the revision setting (8). However, a fair number of studies showed increased rates of transient and permanent recurrent laryngeal nerve injury and hypoparathyroidism following prophylactic central lymph node dissection, even for low volume disease (9). The traditional paradigm assigned the same magnitude of risk for all patients with N1 disease (10). However, small-volume subclinical microscopic N1 disease clearly conveys a much smaller risk of recurrence than large-volume, macroscopic clinically apparent loco-regional metastases (Table 1). With this new information, clinicians will be better able to tailor initial treatment and follow-up recommendations. Implications of N1 stratification for DTC into small-volume microscopic disease versus clinically apparent macroscopic disease importantly relate to issues of prophylactic neck dissection utility, need for pathologic nodal size description, and suggest potential modifications to the AJCC TNM (tumor, nodal disease, and distant metastasis) and American Thyroid Association risk recurrence staging systems (11). Table 2 elaborates on the arguments for and against prophylactic central neck dissection.

## WORKUP AND INITIAL MANAGEMENT

**Figure 1:**
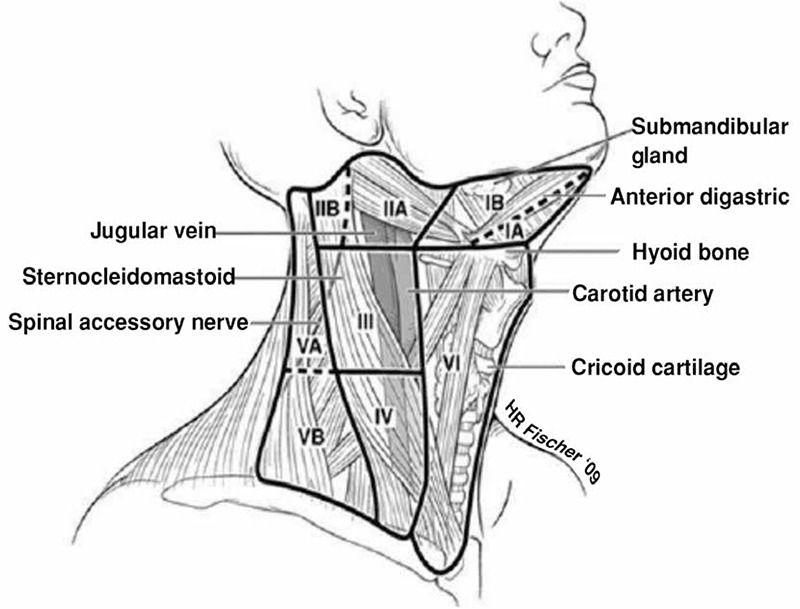
Lymph node compartments of the neck

The primary lymphatic drainage of the thyroid is to the central neck, with subsequent spread to the lower lateral (level IV and V) and then the upper lateral levels, II and III (Figure 1).

Preoperative US is extremely useful for initial staging of cervical lymph nodes. The European Thyroid Association guidelines for cervical ultrasound illustrate very eloquently the sonographic features of lymph nodes that are predictive of malignant involvement as shown in Table 3 (12). Moreover, in experienced hands US may be quite accurate in detecting sub-centimeter metastatic lymph nodes in the lateral neck and even within the central compartment (13,14).

In the presence of lateral neck disease a cross-sectional imaging is warranted. Computerized tomography (CT) provides excellent detail in regard to the local extent of the primary tumor as well as nodal disease in both lateral and central neck and should be utilized when necessary. Fine-needle aspiration (FNAB) biopsy of suspicious lymph nodes should be performed when the clinical and radiological findings are inconclusive and in order to determine the extent of surgery (11). Patients with evidence of nodal disease require therapeutic neck dissection. When disease is limited to the central compartment, clearance of levels VI and VII is recommended. Therapeutic central-compartment neck dissection for patients with clinically involved central nodes should accompany total thyroidectomy to provide clearance of disease from the central neck. This procedure is performed via a horizontal neck incision in a natural skin crease at the lower border of the cricoid cartilage to allow removal of all nodal tissue from the hyoid bone to the innominate artery and from one common carotid artery to the other. The recurrent laryngeal nerves should be carefully dissected and preserved, and the parathyroid glands should be identified and preserved along with their blood supply. When the parathyroid glands are devascularized, they may require auto transplantation in the sternocleidomastoid muscle.

In patients with proven lateral neck disease, therapeutic neck dissection is indicated and can be done in a somewhat selective manner. A rate of metastasis in levels I, IIb and Va are low, and in the absence of proven disease at these levels they can be spared to avoid morbidity, especially to the marginal mandibular and spinal accessory nerves. The lateral neck dissection should normally entail levels IIa through Vb and can be done simply by continuing the thyroidectomy/central neck dissection incision laterally within the same skin crease. In thyroid cancer, special attention should be paid to nodal tissue posterior to the great vessels in level IV, as this is a common site for recurrent nodal disease. Moreover, lymph mode metastases are often found very low in the base of the neck, and dissecting this area may increase the risk for significant vascular and lymphatic injury (15).

Complications of neck dissection can be divided to intraoperative and postoperative complications. These adverse events can further be divided into minor and major. In experienced hands the risk for any complication is between 5%-7% depending on the extent of disease and the risk for major complications is less than 1%. Table 4 summarizes the different complications of lateral and central neck dissection (16).

Studies of the BRAF^V600E^ mutation have suggested an association between presences of the mutation and the risk of nodal disease although results across all patients with DTC are mixed. However, the presence of a BRAF^V600E^ mutation has a limited positive predictive value for recurrence and therefore BRAF^V600E^ mutation status in the primary tumor should not impact on the decision for prophylactic central neck dissection (17).

## FOLLOW UP AND RECURRENT NODAL DISEASE

Neck recurrence in DTC is not an uncommon scenario with up to 30% regional recurrence reported in the literature (18). The risk factors for recurrent nodal disease in the lateral compartment are extra-nodal extension of lymph node metastases and the ratio between positive and excised lymph nodes during the initial neck dissection (19).

This requires thorough assessment of disease and patient-related factors that come into play and based on that decisions can be made. The assessment is usually best done in the context of a multidisciplinary setting. Several reports have shown that most recurrent central compartment nodules do not show clinically significant growth over several years of follow-up and can be safely followed up with serial US (20,21). This approach was subsequently reinforced by the new American Thyroid Association thyroid cancer guidelines for the management of small abnormal cervical lymph nodes (2). Recently, the Thyroid Cancer Care Collaborative (TCCC) published a decision making guide for management of recurrent nodal disease in thyroid cancer and concluded that understanding the biology of DTC allows clinicians to become more conservative in select patients as long as they comprehend the lifelong surveillance of recurrent nodal disease (22).

One of the key elements in the management of regional recurrent DTC is to identify the disease as early as possible. This involves regular surveillance for anyone who has had an operation for thyroid cancer with or without treatment of the neck. There are few modalities useful to detect recurrent disease; one is serum Tg level (23). Stimulated Tg level may be used to produce a more accurate result. However, in many cases a chemical recurrence does not necessarily translate into structural recurrence (24). Routine US is useful in detecting early nodal recurrence but is extremely operator dependent. Nevertheless, in experienced hands it allows quick evaluation of the neck with the option of obtaining FNAB to confirm recurrent nodal disease. In some cases it may be challenging to differentiate central compartment neck recurrence from local recurrence in the thyroid bed. In that setting, obtaining cross-sectional imaging may be of aid. CT scan and MRI should be considered in any case of recurrent nodal disease or in the previously dissected neck when planning a surgical intervention (25). The role of fluorodeoxyglucose positron emission tomography (FDG­PET) is usually limited to less differentiated cancer subtypes, which are usually less RAI avid. These characteristics may sometimes indicate a more aggressive biological behavior of the recurrent tumor. Some tumors may undergo dedifferentiation with increased metabolic activity. These tumors will become more highly FDG-­PET avid as they display less iodine uptake. It has been suggested that this tumor behavior has a negative impact on outcome (26). A recent report concluded that the magnitude of risk for recurrence in patients with N1 disease is not uniform across the board. They found that small-volume subclinical microscopic N1 disease clearly conveys a much smaller risk of recurrence than large-volume, macroscopic clinically apparent loco-regional metastases (27). Patients undergoing revision central compartment dissection for recurrent/persistent disease are at increased risk for vocal fold dysfunction, even when the recurrent laryngeal nerve is anatomically preserved (28). In these cases it is reasonable to closely monitor low volume and sub-centimeter recurrent nodal disease, which in most patients may stay indolent and non-threatening for many years. In the case of progressive nodal disease a selective neck dissection should be performed to remove all structurally apparent disease. This approach prevents unnecessary interventions, which may result in upstaging, increased morbidity and adversely affect quality of life (19).

When gross structural disease is evident and a unilateral/bilateral paratracheal and superior mediastinal dissection is indicated, this should be done in a tertiary care center by an experienced surgeon in order to provide an ontologically safe procedure and achieve minimal morbidity, especially in a setting of prior multiple surgical procedures and/or existing vocal cord paralysis (29,30).

An alternative to surgery or observation of recurrent nodal disease is ultrasound-guided percutaneous ethanol ablation, few institutions in North America and Europe, the most prominent being the Mayo Clinic in Rochester, MN, practice this less conventional method. In their hands this approach is safe and feasible but limited by the number of neck metastases (31).

## CONCLUSION

Lymph node metastases in DTC have a wide spectrum of clinical significance. Several variables are taken under consideration when trying to decide on the optimal management of patients with DTC. Routine prophylactic central and/or lateral lymph node dissection is not advocated with exception of central neck dissection for locally advanced tumors. When regarding recurrent disease, foundations have been laid for clinicians to make accurate decisions as to when to perform surgery and when to continue maintaining the patient’s disease under observation.

These complex decisions are determined based upon multiple factors, not only regarding the patient’s disease but also the patient’s comprehension of the procedure and apprehension levels. Nevertheless if the patient and/or clinician are emotionally keen to surgically remove the disease then the procedure should be considered.

## Figures and Tables

**Table 1:**
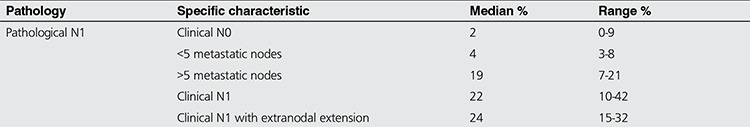
Risk of recurrence based on the characteristics of the cervical lymph node metastases

**Table 2:**
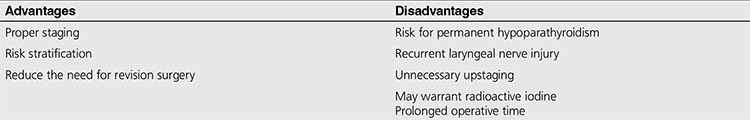
Considerations for and against prophylactic central lymph node dissection

**Table 3:**
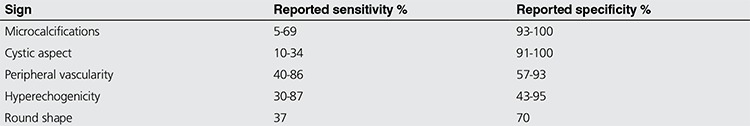
Ultrasound features of lymph nodes predictive of malignant involvement (European Thyroid Association guidelines for cervical ultrasound)

**Table 4:**
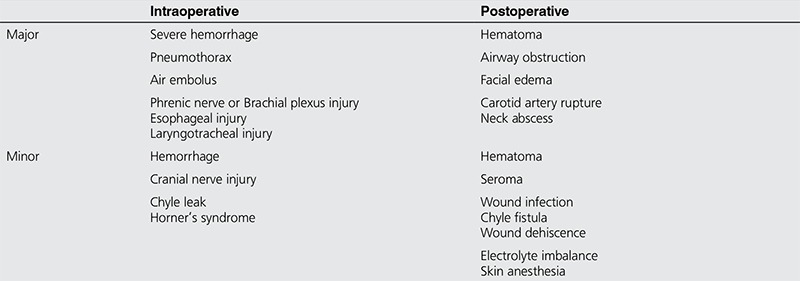
Intraoperative and postoperative complications of neck dissection
